# Successful ventilation through a Rüsch intubation guide catheter in severe laryngotracheal stenosis

**DOI:** 10.1186/s40463-018-0284-9

**Published:** 2018-05-25

**Authors:** Victoria Van Regemorter, Arnaud Potié, Sandra Schmitz, Jean-Louis Scholtes, Laurent Veevaete, Michel Van Boven

**Affiliations:** 1 Department of Anesthesiology, Cliniques Universitaires Saint-Luc, Université catholique de Louvain, Avenue Hippocrate 10, 1200 Bruxelles, Belgium; 2 Department of Head and Neck Surgery, Cliniques Universitaires Saint-Luc, Université catholique de Louvain, Avenue Hippocrate 10, 1200 Bruxelles, Belgium

**Keywords:** Laryngotracheal stenosis, Tracheal surgery, Intubation guide catheter, Ventilation

## Abstract

**Background:**

Providing adequate ventilation may remain complex in patients with severe proximal laryngotracheal stenosis, especially when the airway is shared with the surgeon during tracheal resection surgery. We describe an effective alternative to standard endotracheal intubation using a Rüsch flexible intubation guide catheter.

**Methods:**

In two patients undergoing tracheal repair surgery, we failed to insert a 5.0 inner diameter endotracheal tube (6.9 mm outer diameter) or a 6.0 mm outer diameter endoscope through the laryngotracheal stenosis. However, using indirect laryngoscopy, a 6.0 outer diameter Rüsch flexible intubation guide catheter was passed successfully through the vocal cords and then through the stenosis. Controlled ventilation was achieved by means of the Rüsch guide, provided with its two large Murphy’s eyes. When the trachea was opened, the Rüsch guide was removed just enough for the surgeons to place a Montandon tracheal tube, at that point taking over ventilation. A 7.0 inner diameter endotracheal cuffed tube had been inserted onto the Rüsch guide and left pending upstream from the vocal cords. Once the posterior tracheal wall was sutured, this endotracheal cuffed tube was slid along the Rüsch guide through the vocal cords with the cuff placed beyond the tracheal sutures.

**Results:**

Controlled ventilation through the Rüsch flexible intubation guide catheter showed satisfying and stable ventilatory parameters in both patients. Inspiratory pressures of 25–30 mmHg were enough to reach adequate tidal volumes around 450 ml. End tidal CO_2_ was kept between 35 and 40 mmHg (PaCO_2_ showed similar values). Standard endotracheal intubation at the end of the tracheal resection was easy and safe thanks to the Rüsch guide still in place between the vocal cords.

**Conclusions:**

We suggest an effective and reliable method using a Rüsch flexible intubation guide catheter for airway management in patients suffering from laryngotracheal stenosis in the setting of tracheal repair surgery.

## Background

Airway management in patients with severe laryngotracheal stenosis (LTS) may be challenging for the anesthesiologist, especially when the airway is shared with the surgeon in the context of tracheal resection surgery. Indeed, ensuring adequate ventilation during the initial surgical tracheotomy remains often delicate. Endotracheal intubation through the LTS may not always be possible, even with small size endotracheal tubes (ETT). Keeping the ETT above the LTS is possible, but since these LTS are often situated in the upper part of the airway, the cuff is improperly localized to be inflated. This generates poor mechanical ventilation and airway protection. To overcome this issue we report a safe and effective technique using a Rüsch flexible intubation guide catheter to surgically treat two patients with severe post-intubation LTS.

## Methods

Our alternative method of airway management is described through this retrospective case review of two patients suffering from postintubation LTS and scheduled for tracheal repair surgery. Demographic characteristics of both patients are described in Table 1.

**Table 1:** Demographic characteristics of patients

	Patient 1	Patient 2
Age	75	40
Gender	Female	Female
Weight, height (BMI^a^)	69 Kg, 165 cm (25.3)	63 Kg, 168 cm (22.3)
History of surgery	Thyroidectomy	Uterine cancer surgery
LTS^b^ anteroposterior diameter on CT-scan	7.0 mm	5.2 mm
LTS distance under vocals cords	3 cm	1.5 cm
NYHA^c^ status	Grade III	Grade III
Stridor	Clearly audible	Clearly audible
Respiratory function tests	Limitation in expiratory flow	Limitation in expiratory flow

^a^Body mass index. ^b^Laryngotracheal stenosis. ^c^New York Heart Association

**Fig. 1:**
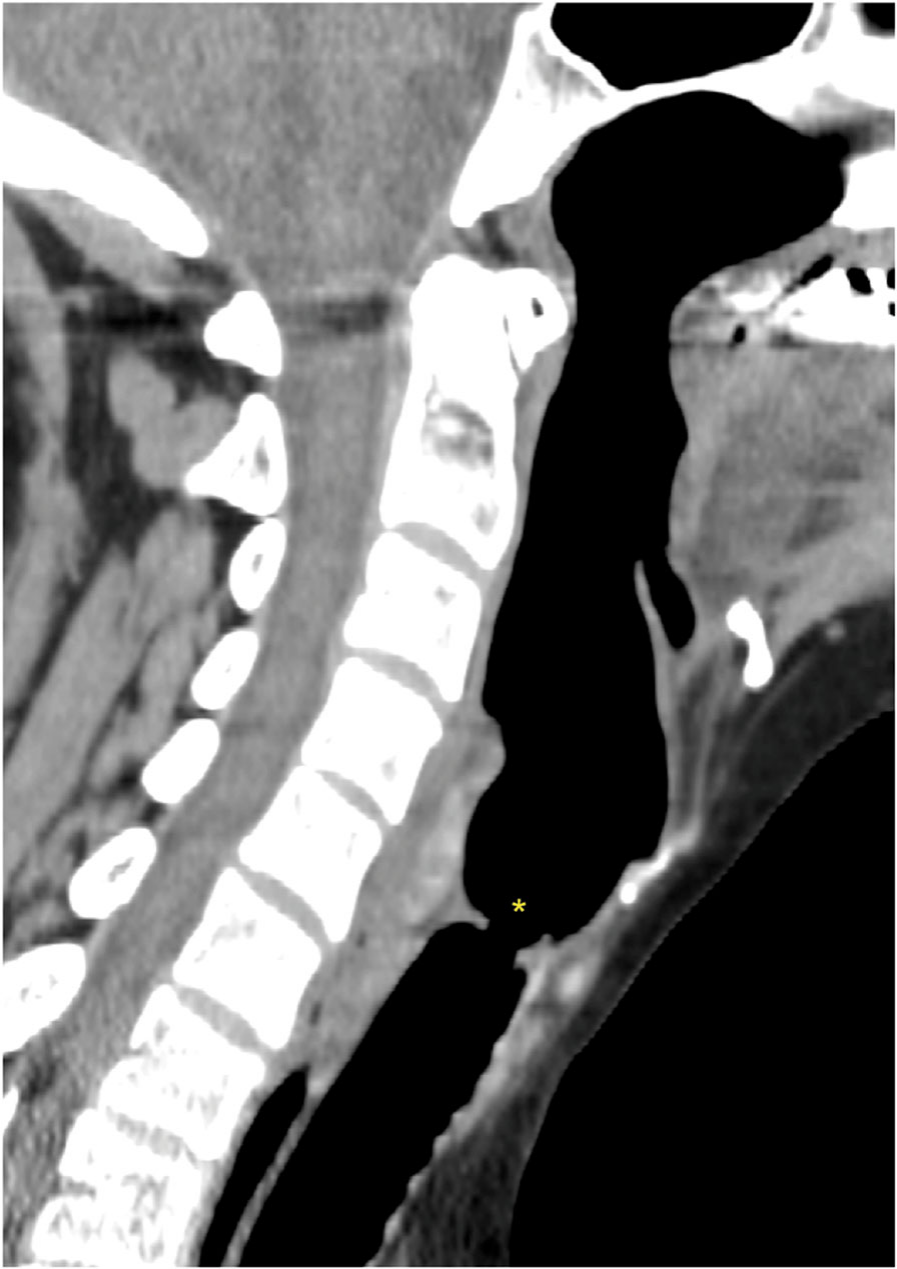
CT-scan longitudinal section of patient 2’s laryngotracheal stenosis (*)

CT-scan longitudinal section of patient 2’s laryngotracheal stenosis is shown in Fig. 1.

Same technique was applied in each case. After preoxygenation, intravenous anesthesia was induced with lidocaine 1 mg.kg^− 1^, titrated propofol 3 mg.kg^− 1^and sufentanil 0.15 μg.kg^− 1^. Manual ventilation went easily. Neuromuscular relaxation was achieved with rocuronium 0.6 mg.kg^− 1^. Indirect laryngoscopy (Airtrak laryngoscope size 2) was used to enhance the visualization of the larynx. In patient 1, a 5.0 mm inner diameter (ID) cuffed microlaryngeal ETT (6.9 mm outer diameter (OD)) (Mallinckrodt™, Ref. 121–50, Covidien llc, Mansfield, MA 02048 USA) failed to pass the LTS. In patient 2, passing an ETT was not even tried since a 6.0 mm OD endoscope had failed to go through the LTS.

**Fig. 2:**
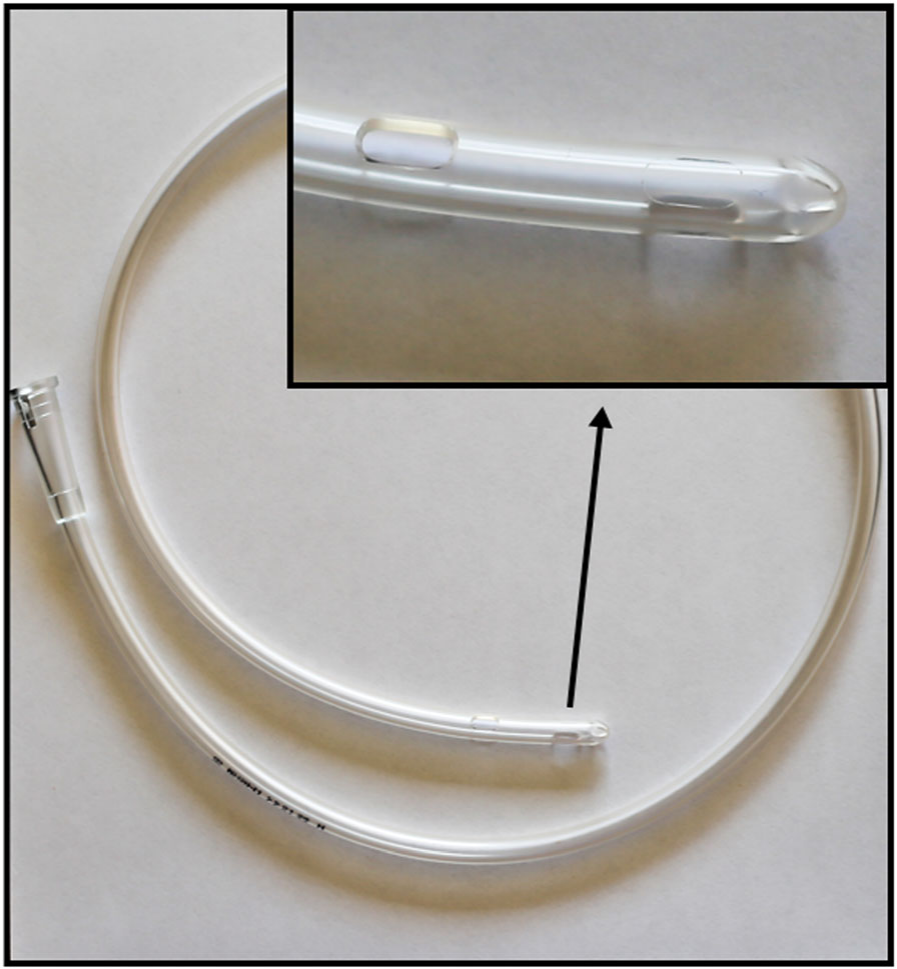
Picture of the Rüsch flexible intubation guide catheter 6.0 outer diameter (OD). The inset shows a close-up view of the distal tip of the Rüsch guide. This tip is equipped with two large Murphy’s eyes and has a rounded atraumatic distal shape

**Fig. 3:**
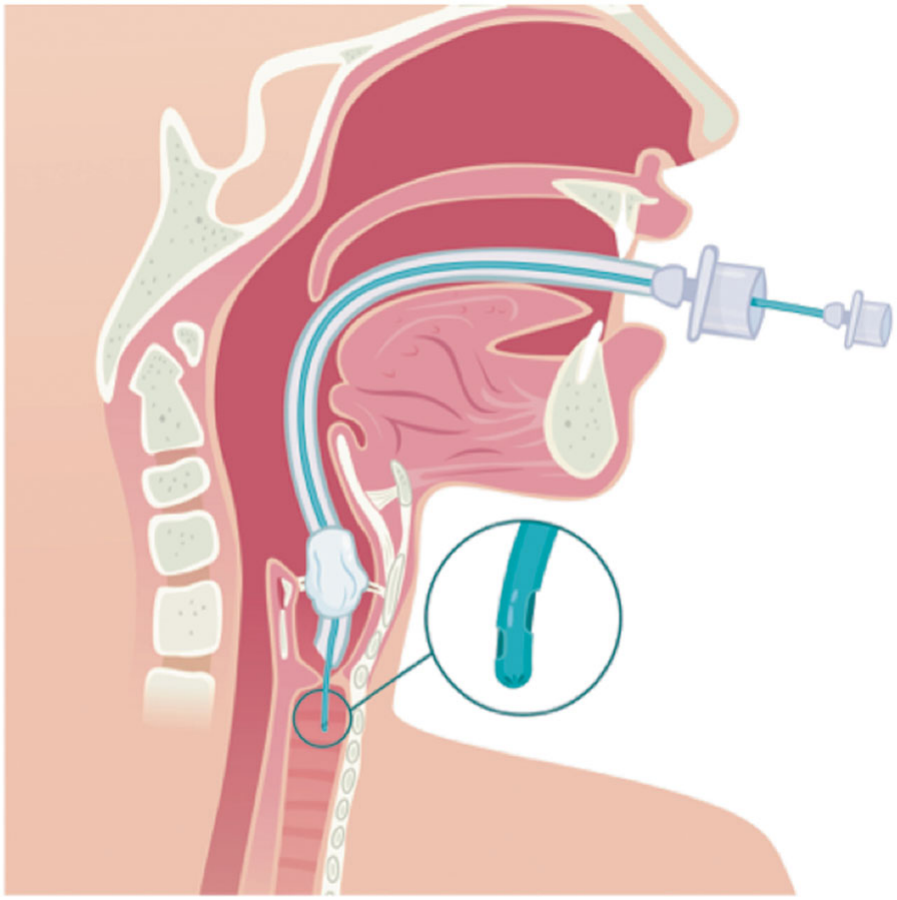
Longitudinal section of upper airways with laryngotracheal stenosis. The Rüsch flexible intubation guide catheter (in green color) is inserted into the endotracheal cuffed tube and is then the only device to successfully pass through the stenosis. Smaller inner diameter (ID) standard connector links the Rüsch guide to the respiratory circuit. Ventilation is made possible thanks to the presence of two large Murphy’s eyes at the extremity of the Rüsch guide

**Fig. 4:**
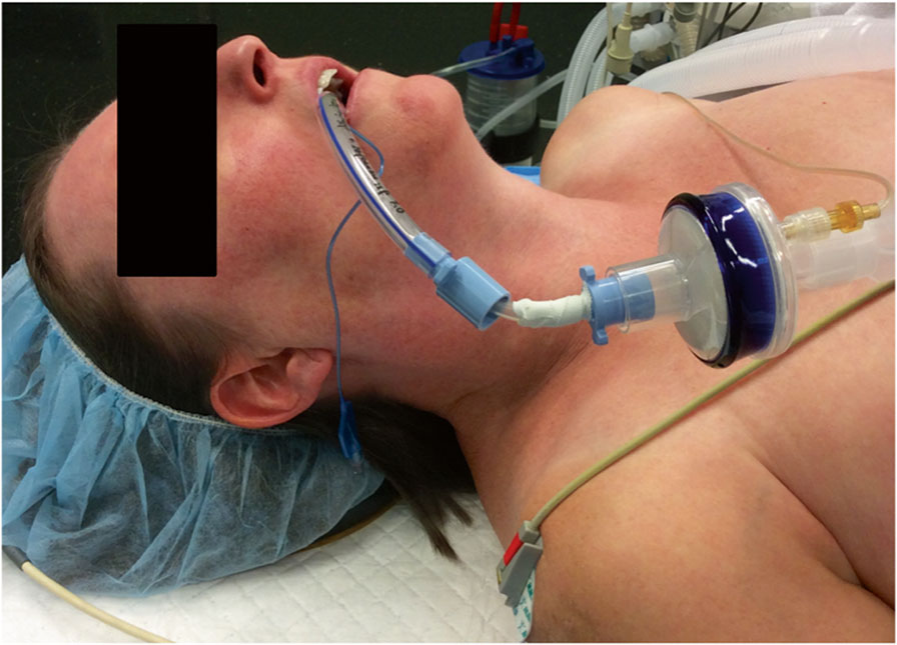
Picture of patient 2. Mechanical ventilation is achieved through the Rüsch flexible intubation guide catheter connected to the breathing circuit. The 7.0 mm inner diameter (ID) endotracheal cuffed tube is left pending upstream from the vocal cords

A Rüsch flexible intubation guide catheter 6.0 mm OD (ERU®, Ref. 225,320, Rüsch Uruguay LTDA, 12100 Montevideo, Uruguay) (Fig. 2) was inserted into a 7.0 mm ID (9.6 mm OD) endotracheal cuffed tube (Portex®, Ref. 100/199/070, Smiths Medical International Ltd., Kent, CT21 6JL, UK). The Rüsch guide was passed through the vocal cords and then pushed softly with rotating movements through the LTS. The ETT was left pending upstream from the vocal cords (Fig. 3 and Fig. 4). The excessive part of the Rüsch guide was cut, leaving a wedge-shaped extremity connected to the filter of the breathing circuit by interposing an ETT 4.5 mm ID standard connector (Portex®, Ref. 100/105/045, Smiths Medical International Ltd., Kent, CT21 6JL, UK). Manual ventilation was easy, pulmonary auscultation and chest expansion were normal and symmetric. Pressure-controlled ventilation was then successfully used. Anesthesia was maintained with sevoflurane. Deep neuromuscular block was ensured with reinjections of rocuronium under close monitoring.

Once trachea was surgically opened downstream from the LTS, the Rüsch guide was removed just enough to allow the surgeons to insert a 7.0 mm ID Montandon tracheal tube (Laryngoflex®, Ref. 121,181, Willy Rüsch GmbH, 71,394 Kernen, Germany) now connected to the breathing circuit. After the resection of the LTS, reanastomosing the tracheal posterior wall was made possible by removing this tube for brief moments. As for the anterior wall suture, the Montandon tube was permanently taken away while the ETT, still pending around the Rüsch guide through the vocal cords, was pushed into the trachea until the surgeons visualized its extremity. Cuff was placed beyond the surgical tracheal sutures to avoid complications such as necrosis or stenosis of the anastomosis. The Rüsch guide was eventually completely removed, the cuff of the ETT was inflated and immediately connected the breathing circuit.

## Results

The trachea was intubated beyond the LTS with a Rüsch flexible intubation guide catheter, connected to the breathing circuit to provide pressure-control ventilation through its two large Murphy’s eyes. Table 2 summarizes ventilatory parameters in our two patients.

**Table 2:** Ventilatory parameters of patients

	Patient 1	Patient 2
Inspiratory pressure (mm Hg)	30	25
Respiratory rate (per minute)	12	11
Inspiratory/expiratory ratio	1/1.5	1/1.5
Tidal volume (ml – ml/kg)	450–6.5	450–7.1
EtCO_2_^a^ (mm Hg)	35–40	35–40
PaCO_2_^b^ (mm Hg)	Not measured	34

^a^End tidal carbon dioxide. ^b^Arterial partial pressure of carbon dioxide

All parameters remained stable during the initial surgical tracheotomy. Afterwards, removing the Rüsch guide just above the LTS was done under direct surgical visualization. Lastly, intubation after tracheal resection was realized easily and safely by sliding forward the pending 7.0 mm ID ETT around the Rüsch guide still in place between the vocal cords.

## Discussion

Airway management in patients with severe LTS undergoing tracheal resection surgery may be complex, especially before tracheotomy. Then, once trachea has been opened, ventilation can be handled either by another sterile ETT placed in the distal trachea or by high frequency jet ventilation (HFJV). Consequently the primary focus of such a surgery is to ensure adequate and safe ventilation of the patient. Several methods for managing this complicated type of airway have been suggested.

Intubating the trachea with an ETT passing through the LTS remains the preferred method [1]. However, it may not be always possible, depending on localization and particularly the severity of the stenosis [2]. ETT could also be placed just ahead of the LTS, but it may result in inadequate ventilation with high peak pressures. Moreover, in our two cases of highly located airway stenosis, the cuff of the ETT would be mislocalized between the vocal folds without any possibility of inflating and thus no airway protection and the risk of ETT dislodgement.

The use of a supraglottic airway (SGA) device has been described in the past [3] and is a common procedure [1], especially using the i-gel [2, 4]. Indeed, SGA devices have the advantage to be easy to insert and to provide ventilation regardless of the localization of LTS. However, it obviously reduces safety in terms of airway protection compared to the use of an ETT without access to the patient’s head. Additionally, other existing methods include dilating the stenosis using serial pediatric ventilating bronchoscopes prior to the insertion of a small ETT [5], using catheter inflation ventilation [6] and providing manual ventilation through a Foley catheter or through airway exchange catheters [7]. A few cases have been reported in spontaneous respiration with bilateral superficial cervical plexus block and dexmedetomidine sedation [8] or with nothing else except a local anesthesia and conscious sedation with midazolam and ketamine [9]. In case of severe LTS, cardiopulmonary bypass might be required [10].

In the present report, we describe the successful use of a Rüsch flexible intubation guide catheter for managing ventilation in patients with severe LTS undergoing tracheal resection surgery. We managed to pass it through two severe LTS while an endotracheal tube of similar OD could not have been possible to use because of its too short length for a standard adult size patient. The Rüsch guide rounded distal tip makes the insertion through a narrow or irregular orifice smooth and atraumatic. Indeed, there was no bleeding or trachea injury. Mechanical ventilation turned out to be quite simple and effective. The presence of a large double Murphy’s eye obviously increases the air flow exit section and cutting short the Rüsch guide (which was achieved easily) also helped decreasing air flow resistance. Hence, the Rüsch guide sets itself apart from the other commercially available intubation catheters because of its relative flexibility, the large double Murphy’s eye and its rounded atraumatic distal tip. So far, there are only two published case reports describing the use of an endotracheal ventilation catheter to ventilate a patient. However, in the first one, HFJV was used [11]. As for the second case report, Cook et al. reported the use of an Aintree intubation catheter as a life saving maneuver to reoxygenate (ventilation conditions not clearly described) a patient extremely difficult to intubate [12]. The risk of HFJV in LTS remains obstruction to exsufflation leading to air trapping. Here, we showed that conventional ventilation through a Rüsch guide provided good oxygenation and ventilation conditions along with satisfying inspiratory pressures and good reliability in regards to end tidal carbon dioxide (EtCO_2_) monitoring. Also, this technique offered a high level of security compared to other techniques described above such as SGA devices. Indeed, the diameter of the Rüsch guide fitted perfectly the stenosis in both cases, which thus ensured better protection against aspiration. In this purpose, a Foley catheter (provided with a cuff) could be superior, but its lack of rigidity compared to the Rüsch guide makes it very hard to manipulate. Lastly, the Rüsch guide was able to fulfill its routine purpose by making the final endotracheal intubation very simple with the final step standard ETT waiting to be blindly but surely pushed into the trachea after resection and anastomosis.

Nevertheless, our method may encounter a few limits. The first one is the size of the LTS since the smallest available Rüsch guide has a 4.0 mm OD (available from 4.0 to 10.0 mm OD). Moreover, achieving mechanical ventilation through a small diameter Rüsch guide (or any standard ETT) can be challenging regarding the pressure regime. Yet, we managed to obtain quite acceptable respiratory pressures in both patients and the same problem can be met with a standard small ETT as well. Of course, the pulmonary condition as well as the BMI of the patient have to be taken into consideration. Another limit is the absence of external markings on the Rüsch guide to facilitate correct depth of insertion into the trachea. Those have to be put manually beforehand and the position checked by chest auscultation.

## Conclusions

We hereby describe a novel method for airway management in patients with severe LTS undergoing tracheal resection surgery. Indeed, Rüsch flexible intubation guide catheters seemed to be reliable devices to use in order to provide proper bilateral lung oxygenation and ventilation, reasonable safety regarding airway protection and ease in handling the different airway management steps in this type of surgery.

## Abbreviations

BMI: Body mass index
EtCO_2_: End tidal carbon dioxide
ETT: Endotracheal tube
HFJV: High frequency jet ventilation
ID: Inner diameter
LTS: Laryngotracheal stenosis
NYHA: New York Heart Association
OD: Outer diameter
PaCO_2_: Arterial partial pressure of carbon dioxide
SGA: Supraglottic airway
